# Evaluation of *Tyrosine Kinase-2* (*TYK2*) signaling pathway gene expression and the presence of the single-nucleotide polymorphism rs12720356 in the peripheral blood of patients with severe psoriasis and loss of systemic treatment response^☆^

**DOI:** 10.1016/j.abd.2025.501165

**Published:** 2025-08-04

**Authors:** Paola Borges Eckstein Canabrava, Roll Stanley Beauge, Samir de Figueiredo Azouz, Renata Velozo Timbó, Luciana Pereira Freire Martins, Bruna Côrtes Rodrigues, Naiara Daris dos Santos, Marcella Palhano Medeiros, Andréa Monteiro de Araújo, Agenor de Castro Moreira dos Santos, Carla Nunes de Araújo, Otávio de Toledo Nóbrega, Patrícia Shu Kurizky, Licia Maria Henrique da Mota, Ciro Martins Gomes

**Affiliations:** a Graduate Program in Medical Sciences, School of Medicine, University of Brasília, Brasília, DF, Brazil; b Graduate Program in Molecular Pathology, School of Medicine, University of Brasília, Brasília, DF, Brazil; c School of Medicine, Federal University of Paraíba, João Pessoa, PA, Brazil

**Keywords:** Biomarkers, Molecular epidemiology, Psoriasis, Therapeutics, TYK2 kinase

## Abstract

**Background:**

RNA sequencing-based studies have identified the transcription processes that contribute to psoriasis development, but the associations of these processes with specific phenotypes need further investigation.

**Objective:**

The authors aimed to determine the associations of specific Peripheral Blood Mononuclear Cell (PBMC) endotypic profiles with loss of treatment response in psoriasis patients.

**Methods:**

A Psoriasis Area and Severity Index (PASI) > 10 was the main outcome. The gene expression of *Tyrosine Kinase-2 (TYK)*, *Interleukin (IL)-12A*, *IL-12B*, *IL-23A*, *IL-23 Receptor (IL-23R)*, *IL-6*, *IL-6R*,*IL-17A* and *Tumor Necrosis Factor (TNF)* in PBMCs was quantified as possible risk factors. Single-Nucleotide Polymorphisms (SNP) were screened using a genotyping technique. Hierarchical clustering of the gene expression results was performed.

**Results:**

The authors included 178 psoriasis patients. *TYK2* was upregulated in the PBMCs of patients with a PASI score > 10, but its distribution was widely variable. A cluster of 19 patients exhibited upregulated expression of most *TYK2*-dependent mediators and increased PASI (p = 0.021) and Dermatology Life Quality Index (DLQI) scores (p = 0.034). Three patients harbored the TYK2I684S variant.

**Study limitations:**

The utility of using single markers for psoriasis diagnosis is limited due to the wide variability of results, but the utility of the simultaneous evaluation of a set of markers has promise.

**Conclusions:**

The present study suggests an association between multiple TYK2 pathway markers and loss of systemic treatment response.

## Introduction

The exact pathophysiology of psoriasis is still unknown and involves a combination of genetic and environmental factors.^1^ One of the key features of psoriasis is the abnormal production of cytokines both systemically and in skin lesions.^2^ Previous studies have identified genes associated with the risk of psoriasis, such as polymorphisms in the genes that encode the *P19* and *p40* subunits of *IL-23* and I*L-12/IL-23*, respectively.^3^, ^4^, ^5^

RNA sequencing (RNA-seq) and microarray techniques have been used to identify various genes involved in transcription-mediated disease processes.^6^, ^7^ However, these techniques may not be effective in detecting wide variations in gene expression.^8^ Therefore, further investigations are needed because the direct relationship between genetic and clinical findings in psoriasis patients is still unknown.^9^, ^10^

Although new medications that target the cytokines involved in psoriasis are available, treatment choices are almost exclusively dependent on the clinical assessment of the disease.^11^, ^12^, ^13^ Biomarkers are measurable signs that can indicate the presence or severity of a disease.^14^ In dermatology, most of these markers are measured in skin samples, but the collection of sequential skin biopsies may be unfeasible.^15^, ^16^, ^17^ In psoriasis patients, many traditional blood markers, particularly those used to detect inflammation (C-reactive protein and erythrocyte sedimentation), have been tested, but the results have been inconclusive.^18^

The primary aim of the current study was to identify specific endotypic profiles of Peripheral Blood Mononuclear Cells (PBMCs) involved in the *Tyrosine Kinase-2* (*TYK2*) signaling pathway and the frequency of the protective psoriasis-related Single-Nucleotide Polymorphism (SNP) rs12720356 and their relationship with the loss of systemic treatment response in psoriasis patients.

## Materials and methods

### Study design and recruitment

This report complies with the Strengthening the Reporting of Observational Studies in Epidemiology – Molecular Epidemiology (STROBE-ME) guidelines.^14^ The study was conducted in a tertiary center for the treatment of patients with psoriasis at the University Hospital of Brasília, which is located in Brasília, Brazil. This center is responsible for a population of more than a million people, and patients are referred from secondary facilities.

From November 1st, 2022, to December 1st, 2023, the authors included consecutive patients attending the Psoriasis Ambulatory Department. To form the main analysis group, the authors included patients who were regularly followed up at the University Hospital of Brasília and who had a confirmed history of severe psoriasis vulgaris (International Classification of Diseases 10th Revision [ICD10 = L40.0]) (Psoriasis Area and Severity Index [PASI > 10] or Dermatology Life Quality Index [DLQI > 10]) and who subsequently achieved disease remission (PASI or DLQI < 5) for at least 1-year after the use of methotrexate or biologics. Consecutive psoriasis patients who were referred to this specialized dermatological center during the same period and who were not receiving systemic treatment were also included to form a comparator group. Twenty healthy controls matched for sex and age to psoriasis patients were also included (convenience sampling) for comparison. The authors excluded patients who had any other autoimmune conditions (except for psoriatic arthritis), pregnant women, or indigenous individuals. All mutation and gene expression tests were conducted on every patient.

After signing an informed consent form, all patients completed a comprehensive questionnaire aimed at gathering detailed clinical information. The information requested included age, sex, time since psoriasis diagnosis and previous medications used for the treatment of psoriasis. All patients were also examined by 2 board-certified dermatologists who collected data related to the type and severity of psoriasis (PASI and DLQI scores).

### Definition of loss of systemic treatment response

In the main analysis group, all psoriasis patients were regularly followed up for more than a year and had previously achieved a satisfactory clinical response to systemic treatment. Upon inclusion in the study, patients were divided into two main groups: 1) Those who developed a loss of systemic treatment response (defined as the recurrence of one or both of the following scores ‒ PASI > 10 or DLQI > 10 ‒ after previously effective treatment) and 2) Those who continued to have a sustained effective response to systemic treatment. Although the group of patients who did not receive any systemic treatment was also divided into patients with a PASI or DLQI > 10 or not, the term “loss of systemic treatment response” was not used for this group.

### Clinical samples

The authors collected 20 ml of whole blood from the study participants in 2 heparin tubes. Immediately after collection, PBMCs were isolated using a Ficoll Paque Plus (Merck KGaA, Darmstadt, Germany) gradient. The samples were stored in RNA Later (Thermo Fisher Scientific, Waltham, United States) at −80 °C until RNA extraction, which was performed up to 1-month after sample collection. Whole blood and plasma were also stored at −80 °C.

### Gene expression analysis

Total RNA from PBMCs was extracted using the mirVana™ PARIS™ RNA and Native Protein Purification Kit (Thermo Fisher Scientific, Waltham, United States). The purified RNA was quantified with a NanoDrop One/OneC UV–vis spectrophotometer (Thermo Fisher Scientific). Samples were treated with RNase-free DNase I (1 U/µl) (Thermo Fisher Scientific). Complementary DNA (cDNA) was generated using a High-Capacity cDNA Reverse Transcription Kit (Thermo Fisher Scientific) in a T100 Thermal Cycler (Bio-Rad, Hercules, United States) according to the manufacturer’s instructions.

Messenger RNA (mRNA) expression analysis was performed using TaqMan-based manufactured probes (Thermo Fisher Scientific) labeled with 5'Fluorescein Amidite (FAM) and 3′Minor Groove Binder (MGB) probes. The authors measured the expression of the *TYK2*, *IL-12A*, *IL-12B*, *IL-23A*, *IL-23* receptor, *IL-6*, *IL-6R*, *IL-17A* and *Tumor Necrosis Factor (TNF)* genes using probes that spanned exon junctions. Despite the primary objective of this study being the evaluation of cytokines in the *IL-23* axis and other activators of the *TYK2* receptors, effector cytokines such as *TNF* and *IL-17A* were additionally measured to assess and compare the activation of the entire Th1 and Th17 stimulation pathway. Three target candidates, namely, *Glyceraldehyde-3-Phosphate Dehydrogenase (GAPDH)*, *eukaryotic 18S rRNA* and *beta actin*, were screened for use as endogenous controls, and *GAPDH* was selected. For each assay, the genes analyzed are detailed in Supplementary Table 1.

Reactions were performed in a QuantStudio 5 Thermocycler (Thermo Fisher Scientific). The reaction was initiated at 50 °C for 2 min, followed by 95 °C for 10 min (polymerase activation) and 40 PCR cycles of 95 °C for 15 s and 60 °C for 60 s. Reactions were performed in a final volume of 15 µl comprising 1× TaqMan™ Gene Expression Master Mix (Thermo Fisher Scientific, Waltham, United States), 1× TaqMan-based probes, 2 µl of cDNA sample from each patient and ultrapure water. All the samples were tested in triplicate. For normalization, the authors used a reference sample (calibrator) formed from a pool of total RNA extracted from the PBMCs of the 20 included healthy controls. No-template controls were also used for comparison.

The authors used the comparative Ct (ΔΔCt) method for data analysis. The individual results are reported as the Relative Quantification (RQ) of gene expression (fold change). Calculations were performed with Applied Biosystems™ Analysis Software (Thermo Fisher Scientific). Values of each expression considered inaccurate are suppressed by the analysis software.

### TYK2 gene variant analyses

The authors chose a robust manufactured assay that precisely detects the psoriasis-related protective Single-Nucleotide Polymorphism (SNP) rs12720356.19 This SNP was the only SNP in the *TYK2* gene that was found to be related to psoriasis and that was reproduced in more than one scientific article, according to a previous systematic review of the literature and meta-analysis.^19^ The kit uses multiplex qPCR with [VIC/FAM] probes to detect the following mutation in the *TYK2* gene: GTGCTCCACGTACTCTGTCACCATG[A/C]TATCTGTAAAGACACAGCTGCTCTG, located on chromosome 19:10359299 on build GRCh38. Transcripts of this region are similar to those of the target tested for *TYK2* gene expression.

Genomic DNA from whole blood was extracted using the PureLink™ Genomic DNA Mini Kit (Thermo Fisher Scientific). The genotyping mixture included 1× TaqPath™ ProAmp™ Master Mix (Thermo Fisher Scientific), 1× TaqMan™ SNP Genotyping (Thermo Fisher Scientific, Waltham, United States) (CN: C_34042925_10, 4351379), 20 ng of genomic DNA and ultrapure water in a final volume of 25 µl. Reactions were performed in a QuantStudio 5 Thermocycler (Thermo Fisher Scientific). The cycling conditions comprised a preread step of 30 s at 60 °C, followed by an initial denaturation/enzyme activation step of 5 min at 95 °C; 40 cycles of denaturation (15 s at 95 °C) and annealing/extension (60 s at 60 °C); and a post read step of 30 s at 60 °C.

### Cytometric bead array assay

The plasma levels of TNF-α, IL-10, IL-1β, IL-8, IL-6 and IL-12p70 in the first 80 included patients were measured using the Human Inflammatory Cytokine Cytometric Bead Array (CBA)-I Kit (RUO) (Becton Dickinson, Franklin Lakes, USA) and a FACSVerse flow cytometer (Becton Dickinson).

### Statistical analysis

For initial statistical analysis, the authors considered the PASI score the main outcome and divided patients into two groups: those with a PASI > 10 and those with a PASI ≤ 10. Another important outcome was the loss of systemic treatment response, defined by a PASI or DLQI score > 10.

The authors explored the differences in the RQs of gene expression and clinical characteristics. Subsequently, the authors applied hierarchical cluster techniques and heatmaps to find groups of patients who could represent a different endotypic profile of individuals experiencing loss of systemic treatment response. The assumptions for multivariate analysis were not met, and this analysis was determined to be unreliable.

The clinical relevance of gene expression changes was defined as a minimum 2× difference in gene expression (RQ) between groups.^20^, ^21^ A p-value < 0.05 indicated statistical significance. Whenever statistical significance was met for RQ, the authors applied Benjamini-Hochberg False Discovery Rate (FDR) adjustment. Statistical analysis was performed using the program R version 4.1.2 (R Core Team [2021]). R: A language and environment for statistical computing. R Foundation for Statistical Computing, Vienna, Austria. URL https://www.R-project.org/).

### Ethics

All patients were included after signing an informed consent form. This study complied with the Declaration of Helsinki and was approved by the Ethics Committee of the Faculty of Medicine of the University of Brasília, Brazil (CAAE: 68068323.3.1001.5558).

## Results

### Clinical profile of the included participants

The authors enrolled 198 participants in this study, including 162 patients with psoriasis who had previously achieved disease remission using systemic medications, 16 patients with psoriasis who were not receiving any systemic treatment, and 20 healthy controls. Twenty-seven psoriasis patients exhibited a PASI score > 10 (the main dependent variable), and another 32 patients exhibited DLQI scores > 10. The data in Supplementary Table 2 show that sex, age and disease duration were not different between patients with PASI scores > 10 and those with PASI scores ≤ 10.

Regarding the medication used by each patient at recruitment, 21 were using methotrexate, 83 were using anti-TNF biologics (5 etanercept, 7 infliximab, and 71 adalimumab), 17 were using anti-IL-12/23 biologics (ustekinumab), 7 were using anti-IL-23 biologics (5 risankizumab and 2 guselkumab), 34 were using anti-IL-17 biologics (secukinumab) and 16 were not using any systemic treatment. The authors did not detect a PASI > 10 in patients treated with anti-IL-23 biologics (n = 7) (Supplementary Table 3).

The percentage of patients who experienced a loss of systemic treatment response (PASI or DLQI score > 10) did not differ among those receiving different systemic treatments (p > 0.05). The number of patients with a PASI or DLQI > 10 was greater among those who did not receive systemic treatment than among patients who received anti-TNF biologics, anti-IL-17 biologics, anti-IL12/23 biologics or anti-IL-23 biologics (p < 0.05) (Supplementary Table 3).

### Gene expression analysis revealed upregulation of TYK2 pathway genes in patients with PASI scores >10

The authors found a positive correlation between *TYK2* gene expression RQ and *IL-12A*, *IL-23A* or *IL-23R* expression (p < 0.05) (Supplementary Fig. 1). Compared with that in the PBMCs of healthy controls, the RQ gene expression of most mediators was similar or reduced in the PBMCs of psoriasis patients. Similar results were found via CBA analysis (Supplementary Tables 4–8).

*TYK2* gene expression in PBMCs was upregulated in patients with a PASI > 10 (Table 1). FDR correction revealed that, in isolation, *TYK2* RQ gene expression could not differentiate patients with a PASI greater than 10 from those with a PASI less than or equal to 10. This result is probably due to the very wide range of RQ gene expression (Supplementary Fig. 2). Additionally, the authors did not find any relationship between *TYK2* gene expression and other demographic or clinical characteristics (Supplementary Table 9).

**Table 1:** Median relative quantification of gene expression (fold change) of mediators according to the psoriasis severity index classification.

Association direction (Clinical relevance)		PASI > 10 × PASI < 10 psoriasis patients				PASI > 10 × PASI <10 psoriasis patients × healthy controls	
		PASI > 10	PASI ≤ 10	p-value	FDR correction	Healthy Controls	p-value^b^
		Median (IQR)	Median (IQR)				
*TYK2*	+	29.23 (66.82)	9.85 (27.92)	0.027^a^	0.243	34.86 (302.45)	0.292
*IL-12A*	‒	26.20 (94.14)	15.58 (40.47)	0.282	0.635	28.63 (160.45)	0.770
*IL-12B*	+	390.30 (2290.73)	116.29 (1275.15)	0.129	0.438	326.67 (574.00)	0.345
*IL-23A*	‒	12.54 (17.71)	11.66 (19.88)	0.741	0.953	36.17 (19.73)	0.838
*IL-23R*	‒	18.14 (25.03)	9.83 (18.67)	0.366	0.659	27.46 (168.96)	0.310
*IL-6*	–	35.096 (229.57)	66.19 (155.49)	0.959	0.959	30.23 (567.87)	0.698
*IL-6R*	–	2.62 (29.65)	1.37 (9.96)	0.146	0.438	1.43 (2.22)	0.165
*TNF-α*	‒	100.41 (337.33)	73.07 (267.18)	0.900	959	203.35 (513.91)	0.911
*IL17-A*	‒	142.92 (1647.96)	77.45 (952.37)	0.465	0.698	137.69 (201.50)	0.345

*TYK2*, *Tyrosine Kinase-2*; IL, Interleukin; R, Receptor; PASI, Psoriasis Area Severity Index; FDR, False Discovery Rate controlled using the Benjamini-Hochberg method; IQR, Interquartile Range; +, Clinically relevant upregulation according to the relative quantification presenting a minimum of 2× positive association; -, Clinically relevant downregulation according to the relative quantification presenting a minimum of 2× negative association; –, No association.

a p < 0.05.

b No significant difference was observed between the gene expression levels of healthy controls compared to psoriasis subgroups divided by the PASI value.

### Hierarchical clustering detected a subgroup of upregulated TYK2 pathway genes

**Fig. 1:**
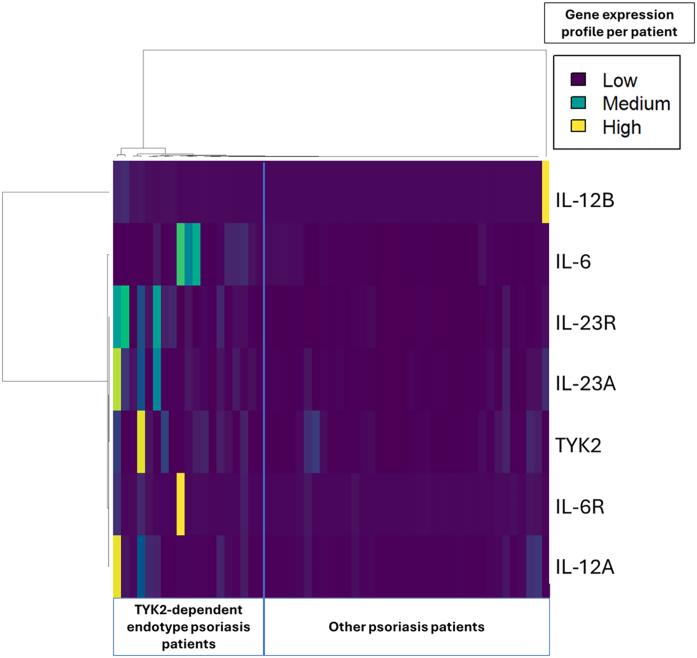
The image displays a heatmap of the gene expression of mediators that belong to the *TYK2* signaling pathway. The data are derived from 55 psoriasis patients who had all the necessary measurements taken in their peripheral mononuclear cells and for whom the replicate results were within 2 standard deviations. This graphic was generated using the program R version 4.1.2 (R Core Team (2021)). R: A language and environment for statistical computing. R Foundation for Statistical Computing, Vienna, Austria. URL https://www.R-project.org/). TYK2, Tyrosine Kinase-2; IL, Interleukin; R, Receptor.

For our hierarchical clustering analysis, the authors included only patients who had RQ values less than 2 standard deviations apart from all mediators tested (Fig. 1). The authors included 55 patients in this analysis (Table 2, Table 3). The authors identified a group of 19 patients with increased expression of mediators of the TYK2 signaling pathway. *IL-12A*, *IL-12B*, *IL-23A*, *IL-23R*, and *IL-6* were upregulated in this group (Table 2). This group of patients also presented higher PASI (p = 0.021) and DLQI (p = 0.034) scores than those with lower *TYK2* mediator expression (Table 3). Patients in this group who exhibited increased expression of mediators of the *TYK2* signaling pathway and also loss of systemic treatment response were using methotrexate, anti-TNF agents and anti-IL-17 biologics but not anti-IL-12/23 or anti-IL-23 biologics (Supplementary Table 10). The remaining patients did not exhibit a gene expression pattern in PBMCs that could distinguish them from other patients (n = 36) (Fig. 1).

**Table 2:** Median relative quantification of gene expression (fold change) of mediators between 2 different endotypic clusters of psoriasis patients identified in this study and healthy controls.

	TYK2- dependent endotype x other psoriasis patients					TYK2- dependent endotype × other psoriasis patients × healthy controls	
	Association direction	TYK2- dependent endotype	Other	p-value	FDR correction		
	(Clinical relevance)	Median (IQR)	Median (IQR)			Healthy Controls	p-value^b^
*TYK2*	+	25.19 (61.98)	10.12 (22.64)	0.150	0.150	34.86 (302.45)	0.241
*IL-12A*	+	23.34 (104.99)	11.44 (20.36)	0.005^a^	0.008^a^	28.63 (160.45)	0.259
*IL-12B*	+	1185.79 (5021.12)	59.25 (216.01)	<0.001^a^	0.002^a^	326.67 (574.00)	0.312
*IL-23A*	+	21.29 (23.0510)	6.52 (8.68)	<0.001^a^	0.002^a^	36.17 (19.73)	0.141
*IL-23R*	+	32.25 (64.88)	6.83 (12.79)	<0.001^a^	0.002^a^	27.46 (168.96)	0.063
*IL-6*	+	163.85 (559.28)	42.52 (115.48)	0.002^a^	0.003^a^	30.23 (567.87)	0.150
*IL-6R*	–	2.45 (9.70)	1.31 (2.98)	0.141	0.150	1.43 (2.22)	0.347
*TNF-α*	+	193.09 (457.85)	39.06 (112.66)	0.091	0.117	203.35 (513.91)	0.199
*IL17-A*		1153.41 (5288.85)	47.47 (228.30)	<0.001	0.002	137.69 (201.50)	0.094

TYK2, Tyrosine Kinase-2; IL, Interleukin; R, Receptor; PASI, Psoriasis Area Severity Index; FDR, False Discovery Rate controlled using the Benjamini-Hochberg method; IQR, Interquartile Range; +, Clinically relevant upregulation according to the relative quantification presenting a minimum of 2× positive association; -, Clinically relevant downregulation according to the relative quantification presenting a minimum of 2× negative association; –, no association.

a p < 0.05.

b No significant difference was observed between the gene expression levels of healthy controls compared to psoriasis subgroups divided by the TYK2-dependent endotype.

**Table 3:** Comparison of demographic and clinical characteristics between the 2 different endotypic clusters of psoriasis patients identified in this study.

	TYK2-dependent endotype	Other psoriasis patients	p-value
**Sex (n)**			0.623
Female	9	21	
Male	10	15	
**Age Median (IQR)**	55.00 (21.50)	46.50 (23.25)	0.190
**Loss of systemic treatment response (n)**^a^	9	10	0.087
PASI > 10 (n)	6	6	0.352
PASI > 5 (n)	10	7	0.026^a^
PASI Median (IQR)	5.6 (8.65)	0.6 (2.50)	0.021^a^
DLQI Median (IQR)	12.00 (15)	1.50 (8)	0.034^a^
Ungual involvement (n)	8	8	0.218
Scalp involvement (n)	6	10	1.000
Genital involvement (n)	5	8	0.749
Palmoplantar involvement (n)	6	10	1.000
Psoriatic arthritis (n)	4	13	0.360
**Total (n)**	19	36	

TYK2, Tyrosine Kinase-2; n, Number of patients; IQR, Interquartile Range; PASI, Psoriasis Area Severity Index.

a p < 0.05.

### Genotyping the TYK2 gene

Three patients (1.69%) were heterozygous for the rs12720356 SNP identified in the *TYK2* gene (Supplementary Fig. 3). This mutation is also known as the *TYK2* I684S variant.^22^ Of the 3 patients carrying the *TYK2* I684S variant, 2 were females, 2 had associated nail psoriasis, and 1 had palmoplantar lesions. All 3 patients had a PASI value < 10. Because the frequency of this SNP was low, it was not possible to relate these findings to the measured gene expression. No mutations were found in healthy controls.

## Discussion

**Fig. 2:**
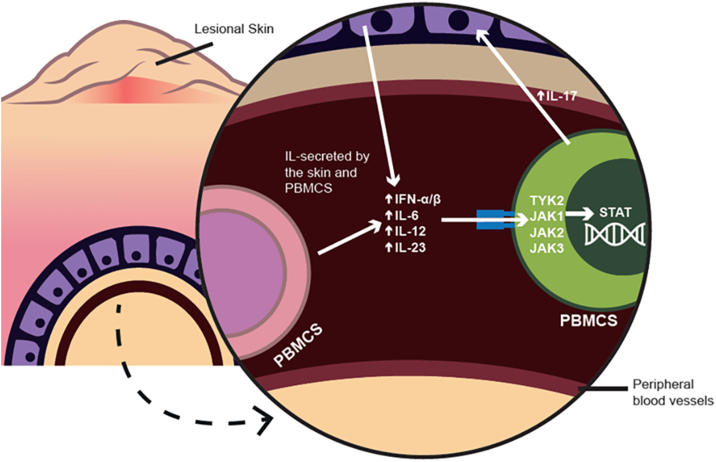
This schematic presents the influence of CD4+ or CD8+ T-cells on peripheral blood mononuclear cells and their influence on the inflammatory loop in psoriasis patients. IL, Interleukin; PBMCS, Peripheral blood mononuclear cells; IFN, Interferon; TYK, Tyrosine kinase; JAK, Janus kinase; STAT, Signal transducer and activator of transcription.

The *TYK2* gene has recently received substantial attention because of its clinical and therapeutic importance in psoriasis.^3^, ^4^ TYK2 is a tyrosine kinase that belongs to the JAK family. The gene is located on chromosome 19p13 and encodes a kinase that promotes *IL-17* transcription via STAT3 phosphorylation, playing a critical role in the pathogenic CD4 T-cell response.^23^ Fig. 2 shows a schematic of the influence of circulating CD4+ or CD8+ T-cells on the inflammatory loop in psoriasis.

The authors quantified the gene expression of mediators involved in the *TYK2* pathway and related them to a specific phenotype of psoriasis. The authors found that compared with healthy controls, psoriasis patients had similar or downregulated *TYK2* pathway RQ gene expression in their PBMCs, regardless of whether they underwent treatment with systemic medications. This systemic downregulation has previously been described in microarray studies including a limited number of patients.^24^ In patients treated with systemic drugs, immunological suppression is expected. However, in patients without systemic treatment, decreased expression of mediators in PBMCs may be a result of increased adhesion of circulating effector mononuclear cells to local skin inflammation, resulting in depletion of these cells.^25^

In the second step, the authors found that *TYK2* gene expression was upregulated in the PBMCs of psoriasis patients with a PASI > 10. However, the range of *TYK2* RQ gene expression was very wide, preventing the use of this marker alone to identify loss of systemic treatment response. Next, the authors used hierarchical clustering to reveal that 19 out of 55 patients evaluated in this step exhibited clear upregulation of genes involved in the TYK2-dependent signaling pathway. These patients had higher PASI and DLQI scores (Table 3). Although the number of patients using anti-IL-23 medications was only 7, it is noteworthy that in the total population, no patient using anti-IL-23 biologics had a PASI greater than 10, although some of them had a DLQI greater than 10. This result is consistent with previous clinical trials that demonstrated an impressive PASI-dependent response in patients using IL-23 blockers.^26^, ^27^

The authors found 3 patients who were heterozygous for a protective psoriasis-related SNP, also known as the TYK2 I684S variant. This variant results in a reduction in phosphorylated (p)-STAT4 levels after IL-12 induction^4^ and in the function of CD4+ and CD8+ T-cells in PBMCs. The frequency of the *TYK2* I684S variant varies from 1% to 9% in the population.^28^ The authors assessed the association between the upregulation of mediators involved in the canonical activation of *TYK2* (*IL-12A, IL-12B, IL-23A, IL-23R, IL-6* and *IL-6R*) and specific phenotypes and identified a cluster of patients with psoriasis who did not use anti-IL-12/23 or anti-IL-23 medications and who exhibited greater PASI scores. Individual markers were not useful for phenotypic psoriasis association, but the utility of the simultaneous evaluation of a set of markers has promise.^29^ Other limitations of the study must be acknowledged, including the potential for confounding factors, which are an inevitable concern in any observational study. While such factors could indeed cause worry, all subgroup analyses conducted in this article indicated a significant upregulation of the inflammatory TYK2-dependent pathway in patients who exhibited a loss of response to systemic treatments.

Although this study is one of the largest of its kind to explore complex gene expression analysis in the blood of psoriasis patients, the relatively modest sample size, particularly among patients using IL-23 blockers, must be taken into account. Nonetheless, the findings presented here underscore the necessity for future clinical trials to investigate the efficacy of TYK2 inhibition in bio-experienced psoriasis patients, especially those currently using the most effective biological drugs available on the market. These trials could potentially pave the way for more tailored and efficacious treatment options, bringing renewed hope to patients grappling with this challenging condition.

Recent treatment developments for psoriasis have focused on the TYK2-dependent immunological response. Deucravacitinib is an oral, selective, allosteric TYK2 inhibitor that is licensed for plaque psoriasis treatment in some countries.^30^ Our study revealed that TYK2 pathway activity may be upregulated in patients who do not respond to anti-TNF and anti-IL-17 biologics. This finding suggests that deucravacitinib could be a promising option for patients who require second or third-line treatment.

The successful relation of previously described genotypes to the clinical phenotypes of a group of psoriasis patients who experienced a loss of systemic treatment response reinforces the importance of simultaneously assessing multiple molecular markers for the development of new tools for the evaluation of psoriasis patients.

## Editor

Luciana P. Fernandes Abbade.

## Financial support

This work was supported by the Fundação de Apoio a Pesquisa do Distrito Federal No. 00193-00000279/2023-70, Brazil. The funders had no role in the study design, data collection and analysis, decision to publish, or preparation of the manuscript.

## Authors' contributions

Paola Borges Eckstein Canabrava:

Conceptualization, Investigation.

Roll Stanley Beauge:

Investigation.

Samir de Figueiredo Azouz:

Investigation.

Renata Velozo Timbó:

Software, Validation, Formal analysis.

Luciana Pereira Freire Martins:

Software, Validation, Formal analysis.

Bruna Côrtes Rodrigues:

Software, Validation.

Naiara Daris dos Santos:

Investigation.

Marcella Palhano Medeiros:

Investigation, Validation.

Andréa Monteiro de Araújo:

Data curation.

Agenor de Castro Moreira dos Santos:

Methodology.

Carla Nunes de Araújo:

Investigation, Software, Formal analysis.

Otávio de Toledo Nóbrega:

Investigation, Software, Formal analysis.

Patrícia Shu Kurizky:

Investigation.

Licia Maria Henrique da Mota:

Methodology, Resources.

Ciro Martins Gomes:

Investigation, Supervision, Project administration.

## Research data availability

The entire dataset supporting the results of this study was published in this article.

## Conflicts of interest

None declared.
