# Supplementary material for: Evaluation of Tyrosine Kinase-2 (TYK2) signaling pathway gene expression and the presence of the single-nucleotide polymorphism rs12720356 in the peripheral blood of patients with severe psoriasis and loss of systemic treatment response
Source: An Bras Dermatol. 2025 Aug 4;100(5):501165. doi: 10.1016/j.abd.2025.501165 (PMC12539401; doi:10.1016/j.abd.2025.501165)
Supplement: Supplementary file 1 [file mmc1.pdf]

ABD-D-24-00680_ Supplementary Material

Supplementary Table 1 Complete information about the manufactured TaqMan assays (Thermo Fisher Scientific, Waltham, United States) used in this study.

Gene symbol	Gene name	Catalog number	Gene aliases	Human chromosome location
TYK2	Tyrosine kinase 2	Hs00177464_m1	IMD35, JTK1	Chr.19: 10350528-10380572 on build GRCh38
IL-12A	Interleukin 12A	Hs01073447_m1	CLMF, IL-12A, NFSK, NKSF1, P35	Chr.3: 159988836-159996019 on build GRCh38
IL-12B	Interleukin 12B	Hs01011518_m1	CLMF, CLMF2, IL-12B, IMD28, IMD29, NKSF, NKSF2	Chr.5: 159314783-159330473 on build GRCh38
IL-23A	Interleukin 23 subunit alpha	Hs00372324_m1	IL-23, IL-23A, IL23P19, P19, SGRF	Chr.12: 56334159-56340410 on build GRCh38
IL-23R	Interleukin 23 receptor	Hs00332759_m1	–	Chr.1: 67138638-67259979 on build GRCh38
IL-6	Interleukin 6	Hs00174131_m1	BSF-2, BSF2, CDF, HGF, HSF, IFN-beta-2, IFNB2, IL-6	Chr.7: 22725889-22732002 on build GRCh38
IL-6R	Interleukin 6 receptor	Hs01075664_m1	CD126, IL-6R-1, IL-6RA, IL6Q, IL6RA, IL6RQ, gp80	Chr.1: 154405193-154469450 on build GRCh38

TNF	Tumor necrosis factor	Hs00174128_m1	DIF, TNF-alpha, TNFA, TNFSF2, TNLG1F	Chr.6: 31575567-31578336 on build GRCh38
IL-17A	Interleukin 17A	Hs00174383_m1	CTLA-8, CTLA8, IL-17, IL-17A, IL17	Chr.6: 52186387-52190638 on build GRCh38
GAPDH	Glyceraldehyde-3-phosphate dehydrogenase	Hs99999905_m1	G3PD, GAPD, HEL-S-162eP	Chr.12: 6534405-6538375 on Build GRCh38

Supplementary Table 2 Demographic characteristics of all the psoriasis patients included, according to the psoriasis area severity index classification.

	**PASI >10 (n=27)**	**PASI ≤10 (n=151)**	**Total**	**p-value**
**Sex (n)**				0.196
F	9	74	83	
M	18	77	95	
**Age Median (IQR)**	46 (22)	50(21)	49 (21.75)	0.257
**BMI Median (IQR)**	29.91 (6.20)	28.12 (4.89)	28.39 (5.13)	0.163
**Disease duration (years) Median (IQR)**	19.44 (12.17)	19.19 (11.06)	19.23 (11.20)	0.921
DLQI Median (IQR)	13 (9.50)	2 (8.00)	3 (11)	<0.001*
Ungual involvement (n)	17	28	45	<0.001*
Scalp involvement (n)	23	45	68	<0.001*
Genital involvement (n)	15	26	41	<0.001*
Palmoplantar involvement (n)	1	13	14	0.698
Psoriatic arthritis (n)	6	35	41	1.000

PASI, Psoriasis Area and Severity Index; n, Number of patients; IQR, Interquartile Range; BMI, Body Mass Index; DLQI, Dermatology Life Quality Index.

Supplementary Table 3 Treatment characteristics of the psoriasis patients included, according to the psoriasis area severity index and loss of systemic treatment response classification.

	**Loss of systemic treatment response**					
	**PASI >10**	**PASI ≤10**	**p-value**	**PASI or DLQI >10**	**PASI OR DLQI ≤10**	**p-value**
**Treatment class (n)**			0.001			<0.001
Methotrexate	3	18		9	12	
Anti-TNF	7	76		17	66	
Anti-IL12/23	2	15		5	12	
Anti-IL23	0	7		3	4	
Anti-IL17	6	28		13	21	
**No systemic treatment**	9	7		12	4	

Total	27	151		59	119	

n, number of patients; PASI, Psoriasis Area and Severity Index; TNF, Tumor Necrosis Factor; IL, Interleukin. PASI < 10 statistical comparisons: methotrexate × no systemic treatment, p = 0.047; anti-TNF × no systemic treatment, p < 0.001; anti-IL12/23 × no systemic treatment, p = 0.048; anti-17 × no systemic treatment, p = 0.047. PASI or DLQI > 10 (loss of systemic treatment response) statistical comparisons = anti-TNF × no systemic treatment, p < 0.046; anti-IL12/23 × no systemic treatment, p = 0.015; anti-IL17 × no systemic treatment, p = 0.032; anti-IL23 × no systemic treatment, p = 0.018.

Supplementary Table 4 Median relative quantification gene expression (fold change) of all psoriasis patients compared to that of healthy controls.

	Association direction	Psoriasis patients	Healthy controls		FDR
		Median (IQR)	Median (IQR)	p-value	correction
**TYK2**	-	10.72 (40.58)	34.86 (302.45)	0.066	0.203
**IL-12A**	–	17.21 (48.15)	28.63 (160.45)	0.116	0.203
**IL-12B**	–	184.82 (1705.10)	326.67 (574.00)	0.463	0.648
**IL-23A**	-	11.76 (21.08)	36.17 (19.73)	0.109	0.203
**IL-23R**	-	11.50 (27.06)	27.46 (168.96)	0.031[a]	0.203
**IL-6**	+	62.06 (159.06)	30.23 (567.87)	0.952	0.952
**IL-6R**	–	1.690 (6.48)	1.43 (2.22)	0.837	0.952

TYK2, Tyrosine Kinase-2; IL, Interleukin; R, Receptor; FDR, False Discovery Rate controlled using the Benjamini-Hochberg method; IQR, Interquartile Range; +, Clinically relevant upregulation according to the relative quantification presenting a minimum of 2× positive association; -, Clinically relevant downregulation according to the relative quantification presenting a minimum of 2× negative association; –, No association.

Supplementary Table 5 Median relative quantification gene expression (fold change) of psoriasis patients without systemic treatment compared to that of healthy controls.

	Association direction	Psoriasis patients without systemic treatment	Healthy controls	
		Median (IQR)	Median (IQR)	p-value

**TYK2**	–	33.29 (36.54)	34.86 (302.45)	0.485
**IL-12A**	–	28.63 (128.07)	44.04 (160.45)	1.000
**IL-12B**	+	1378.45 (2586.35)	326.67 (574.01)	0.394
**IL-23A**	+	35.18 (18.72)	11.60 (21.08)	0.309
**IL-23R**	–	29.83 (174.14)	27.46 (168.96)	0.485
**IL-6**	-	8.22 (30.92)	30.24 (567.87)	0.413
**IL-6R**	+	13.07 (10.86)	1.42 (2.22)	0.286

TYK2, Tyrosine Kinase-2; IL, Interleukin; R, Receptor; FDR, False Discovery Rate controlled using the Benjamini–Hochberg method; IQR, Interquartile range; +, Clinically relevant upregulation according to the relative quantification presenting a minimum of 2× positive association; -, clinically relevant downregulation according to the relative quantification presenting a minimum of 2× negative association; –, No association.

Supplementary Table 6 Median quantification in picograms per milliliter (pg/mL) of cytometric bead array results for plasma samples from psoriasis patients divided according to the psoriasis area severity index classification.

	Association direction	PASI >10	PASI ≤10	
		Median (IQR)	Median (IQR)	p-value
**TNF**	–	5.92 (0.40)	5.99 (0.63)	0.439
**IL-12p70**	–	4.64 (0.61)	5.02 (0.45)	0.181
**IL-6**	–	7.69 (3.70)	6.72 (2.04)	0.086
**IL-1β**	–	10.03 (0.53)	10.07 (0.74)	0.237
**IL-8**	–	18.50 (13.94)	15.85 (7.57)	0.271
**IL-10**	–	3.65 (0.71)	3.49 (0.47)	0.799

PASI, Psoriasis Area Severity Index; TNF, Tumor Necrosis Factor; IL, Interleukin; IQR, Interquartile Range; +, Clinically relevant positive association according to the relative quantification presenting a minimum of 2× positive association; -, Clinically relevant negative association according to the relative quantification presenting a minimum of 2× negative association; –, No association.

Supplementary Table 7 Median quantification in picograms per milliliter (pg/mL) of cytometric bead array results for plasma samples from psoriasis patients versus those from healthy controls.

	Association direction	Psoriasis patients	Healthy controls	p-value	FDR correction
		Median (IQR)	Median (IQR)		
**TNF**	-	5.94 (0.61)	24.17 (61.64)	0.008	0.024[a]
**IL-12p70**	–	5.02 (0.52)	4.88 (0.71)	0.902	0.992
**IL-6**	–	6.76 (2.38)	6.72 (0.51)	0.992	0.992
**IL-1β**	–	10.07 (0.63)	10.47 (1.19)	0.126	0.252
**IL-8**	-	16.49 (7.75)	119.31 (187.26)	0.005	0.024[a]
**IL-10**	–	3.52 (0.47)	3.68 (0.26)	0.518	0.777

TNF, Tumor Necrosis Factor; IL, Interleukin; IQR, Interquartile Range; +, Clinically relevant positive association according to the relative quantification presenting a minimum of 2× positive association; -, Clinically relevant negative association according to the relative quantification presenting a minimum of 2× negative association; –, No association.

Supplementary Table 8 Median quantification in picograms per milliliter (pg/mL) of the cytometric bead array results for plasma samples from psoriasis patients without systemic therapy versus those from healthy controls.

	Association direction	Psoriasis patients without systemic treatment	Healthy controls	p-value
		Median (IQR)	Median (IQR)	
**TNF**	-	6.06 (0.96)	24.17 (71.74)	0.069
**IL-12p70**	–	5.22 (0.71)	4.88 (0.28)	0.915
**IL-6**	–	7.69 (1.34)	6.72 (0.51)	0.476
**IL-1β**	–	10.10 (0.29)	10.47 (1.19)	0.069
**IL-8**	-	18.59 (4.17)	119.31 (187.26)	0.067
**IL-10**	–	3.45 (0.21)	3.67 (0.26)	0.392

TNF, Tumor Necrosis Factor; IL, Interleukin; IQR, Interquartile Range; +, Clinically relevant positive association according to the relative quantification presenting a minimum of 2× positive association; -, Clinically relevant negative association according to the relative quantification presenting a minimum of 2× negative association; –, No association.

Supplementary Table 9 Comparison of the median relative quantification gene expression (fold change) of tyrosine kinase 2 according to the demographic and clinical characteristics of psoriasis patients.

	Association direction	Yes	No	p-value
		Median (IQR)	Median (IQR)	
**Female sex**	–	16.66 (60.66)	7.97 (22.67)	0.131
**Ungual involvement**	–	22.51 (59.53)	10.11 (28.28)	0.288
**Scalp involvement**	–	10.76 (23.41)	10.69 (43.29)	0.719
**Genital involvement**	–	10.43 (16.54)	10.77 (42.86)	0.618
**Palmoplantar involvement**	–	5.22 (62.71)	10.77 (38.21)	0.399
**Psoriatic arthritis**	–	10.01 (57.91)	10.76 (33.32)	0.725

IQR, Interquartile Range.

Supplementary Table 10 Comparison of the treatments used by patients with the 2 different endotypic clusters identified in this study.

	TYK2-dependent endotype	Other
	Loss of systemic treatment response, n (total)	Loss of systemic treatment response, n (total)
**Methotrexate**	2 (3)	0 (2)
**Anti-TNF**	2 (5)	6 (21)
**Anti-IL12/23**	0 (1)	0 (3)
**Anti-IL23**	0 (0)	0 (1)
**Anti-IL17**	5 (8)	4 (8)
	**PASI or DLQI >10**	**PASI or DLQI >10**
	**n (total)**	**n (total)**
**No systemic treatment**	2 (2)	1 (1)

n, Number of patients; TYK2, Tyrosine Kinase-2; TNF, Tumor Necrosis Factor; IL, Interleukin; PASI, Psoriasis Area Severity Index; DLQI, Dermatology Life Quality Index.

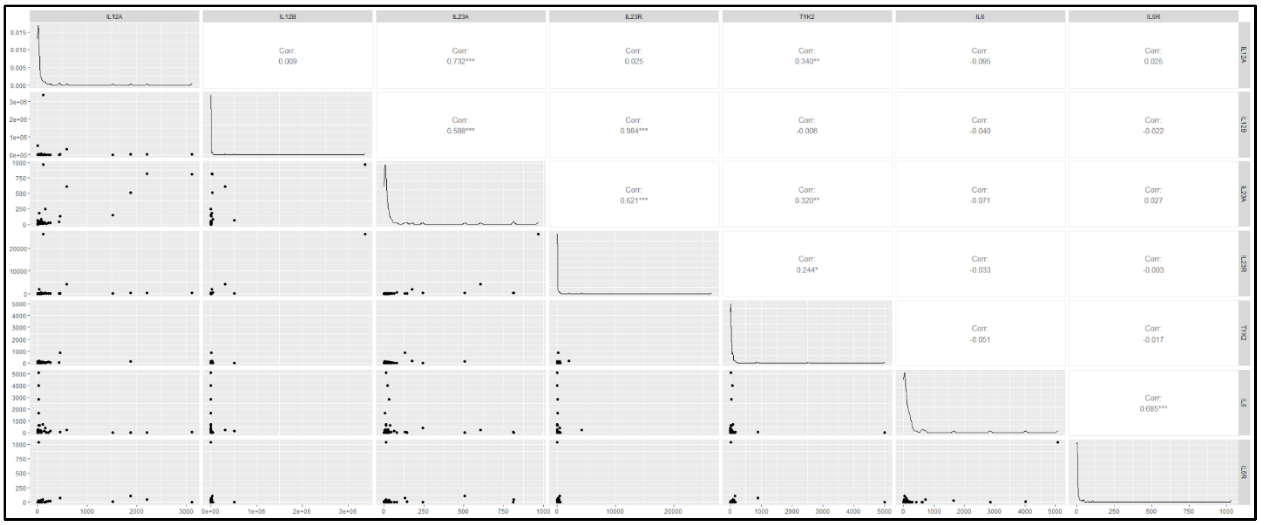

Supplementary Figure 1 A correlation scatter plot showing gene expression associations between all mediators examined in the study. The results showed a positive correlation between Tyrosine Kinase 2 (TYK2) gene expression and Interleukin (IL) 12A, IL-23A and the IL-23 Receptor (R), which are involved in the canonical activation pathway of the TYK2 receptor. Positive or negative values represent positive or negative correlations, respectively. Corr, Correlation, *p<0.05, **p<0.01, ***p<0.001. This graphic was generated using the program R version 4.1.2 (R Core Team (2021). R: A language and environment for statistical computing. R Foundation for Statistical Computing, Vienna, Austria. URL https://www.R-project.org/).

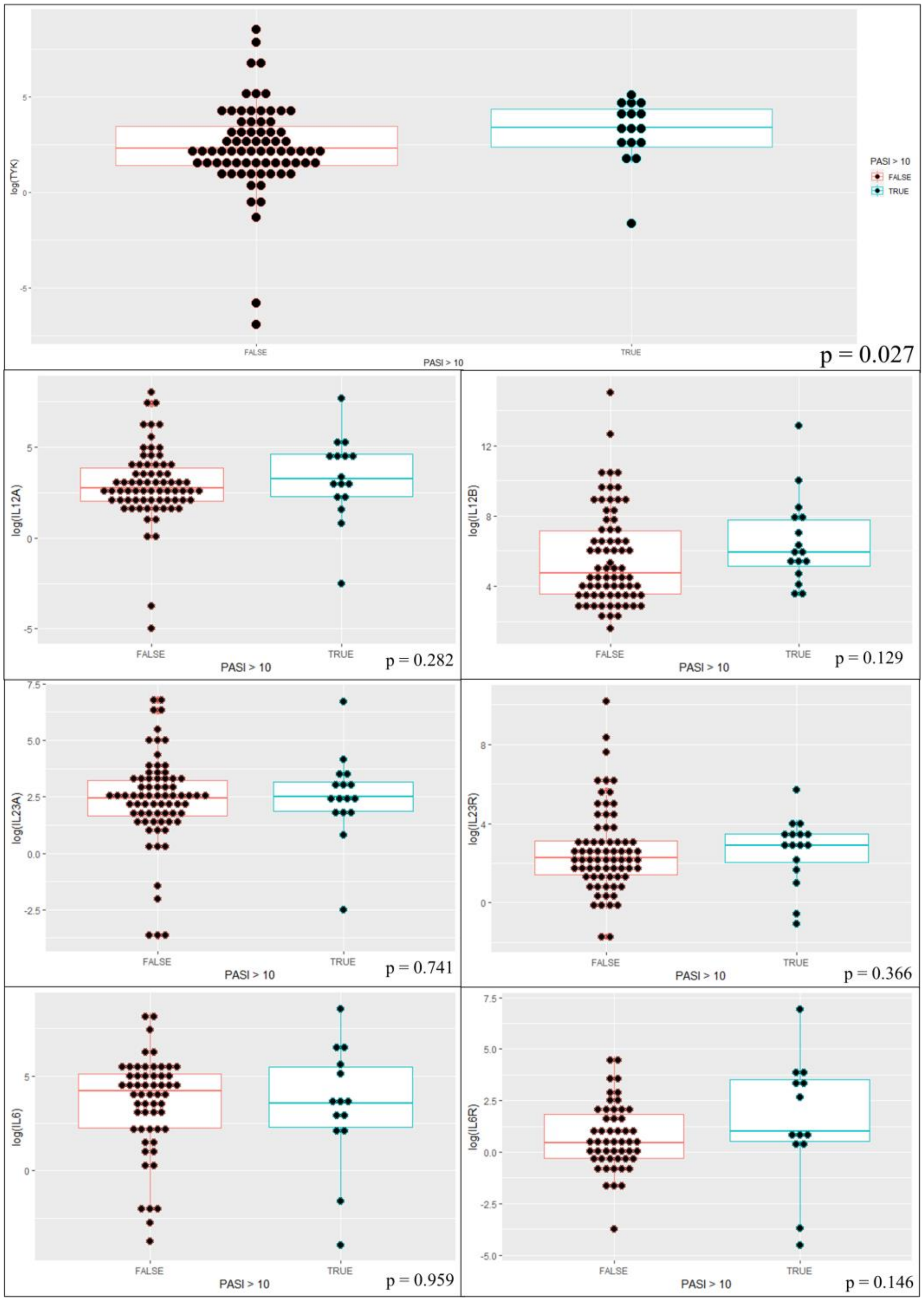

Supplementary Figure 2 A dot plot showing the logarithmic relative quantification gene expression (fold change) values for all patients who were evaluated for psoriasis. The median and quartiles of patients are presented, grouped by their Psoriasis Area and Severity Index (PASI). TYK2, Tyrosine Kinase 2; IL, Interleukin; R, Receptor; PASI, Psoriasis Area Severity Index.

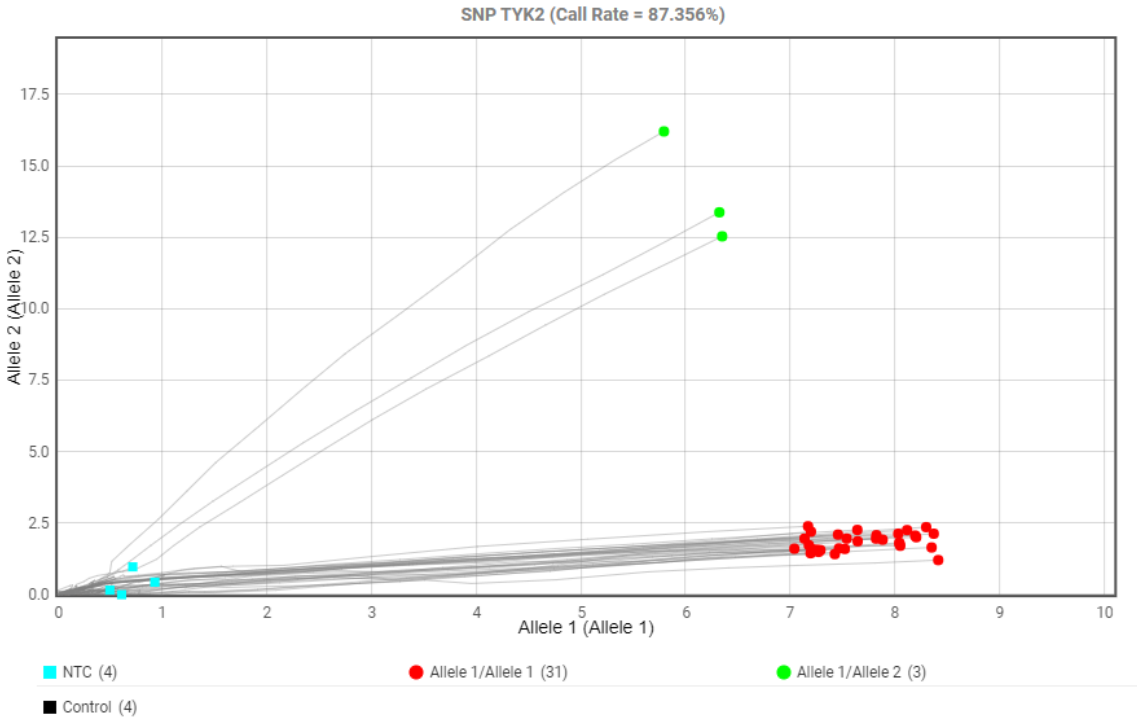

Supplementary Figure 3 A figure showing the results of genotyping tests: 3 patients (1.69%) with a heterozygous mutation for the single-nucleotide polymorphism rs12720356 (the TYK2 I684S variant) that is protective against psoriasis. NTC, No-Template Control. Calculations were performed with Applied Biosystems™ Analysis Software (Thermo Fisher Scientific).
